# Epstein-Barr virus associated gastric carcinoma: *a report from Iran in the last four decades*

**DOI:** 10.1186/1746-1596-2-25

**Published:** 2007-07-15

**Authors:** Afshin Abdirad, Siavash Ghaderi-Sohi, Karem Shuyama, Chihaya Koriyama, Hosain Nadimi-Barforoosh, Sara Emami, Alireza Mosavi-Jarrahi, Azin Nahvijou, Suminori Akiba

**Affiliations:** 1 The Cancer Research Center, the Cancer Institute, Tehran University of Medical Sciences, Imam Khomeini Medical Center, Keshavarz Blvd., Tehran, Iran; 2 Department of Epidemiology and Preventive Medicine, Kagoshima University Graduate School of Medical and Dental Sciences, Japan

## Abstract

**Background:**

Epstein-Barr virus has been proved to be associated with many of the human malignancy including gastric carcinoma, one of the most important human malignancies in the world. There has been no study about the presence of EBV in gastric adenocarcinoma in Iran.

**Methods:**

We examined the presence of EBV in 273 formalin fixed paraffin-embedded cases of gastric carcinoma from Cancer institute of Tehran University, from 1969 to 2004. In situ hybridization of EBV-encoded small RNA-1 (EBER-1) was conducted. The strain of positive cases was examined by means of polymerase chain reaction and/or restriction fragment length polymorphism analysis.

**Results:**

We found 9 (3%; 95% CI = 1–5%) EBV positive cases. The gender difference was not statisticaly significant. The proportion of EBV-GC cases in diffuse type was higher than intestinal type (OR = 0.08; 95% CI = 0.002–0.64). EBV-GC cases had no relation with age, location and invasion. Six out of 9 EBV-GC cases were born during the period between 1928 and 1930. All 9 cases were Type A. Prototype F was seen in 6 out of 8 cases. Type "i" was found in 8 cases and type I in 1 case. XhoI+ and XhoI- polymorphism accounted 6 and 3 of the cases, respectively.

**Conclusion:**

Our study is the first to describe the frequency of EBV-GC in Iran and the Middle East, highlighting a very low prevalence with specific clinicopathologic features. The predominance of EBV-GC birth year in a fixed period, suggests that EBV infection or other events at early childhood may be related to the development of EBV-GC later in the life. The predominance of the type "i" and XhoI+ cases are contradictory to other studies in Asia and is similar to what is reported from Latin American countries.

## Background

Epstein-Barr virus (EBV) is a ubiquitous double stranded DNA virus from human herpes virus family, which has B-lymphotropism [1]. More than 90% of adults in the world have serologic evidence of infection with this virus [1]. It is acquired during early childhood and the age of infection is much lower in undeveloped countries with low socioeconomic condition [1]. Following primary infection, the virus establishes a life-long latent infection in the B-cell lymphocytes where it is present in 1 in 10^5^–10^6 ^circulating cells [2]. It expresses some antigens in this latent phase, which are proved to have some oncogenic properties [3-8]. There are many documents about causal association between EBV and lymphoid malignancies such as Burkitt's lymphoma in equatorial Africa [9], nasal T/NK cell lymphoma [10], Hodgkin's lymphoma [11] and B-cell lymphoma in immunosuppressed patients [12]. EBV has also the capacity of infecting epithelial cells, and also has tumorogenic effect in these cells from which the nasopharyngeal carcinoma is one of the important prototypes [13].

The first report of EBV involvement in gastric carcinoma was published in 1990 in lymphoepithelial carcinoma of the stomach [14]. Consequently, in 1992 Shibata and Weiss reported EBV detection in 16% of ordinary gastric carcinoma in U.S. [15]. Since then, the prevalence of EBV-associated gastric carcinoma (EBV-GC) has been investigated and reported in various countries which ranged between 3 to 18% [15-34] with the lowest prevalence in Papua New Guinea, Pakistan and Korea which is between 1 to 3% and the highest in Germany and U.S. which is 16 to 18%.

There are many evidences of causal relationship between EBV and gastric carcinoma such as: monoclonality of the viruses in neoplastic cells which was demonstrated by unique terminal repeat of EBV DNA [35]; uniform presence of EBV in all tumoral cells detected by in situ hybridization of EBER-1, but not in surrounding epithelial cells [15-34] and elevation of IgG and IgA antibodies against viral capsid antigen several months before clinical presentation of the disease [36].

EBV-GC shows some constant characteristic clinicopathologic features such as predisposition to upper two-third of the stomach [15-17,19-31]; a moderately differentiated tubular to poorly differentiated solid type of histology [16-22,32,33]; having no effect in stromal invasion and survival [15,16]; and male predominance in most of the studies. However, the last one is not seen in some exceptional studies which are performed in Mexico [32,37], Chile [16] and China [38]. Age dependence is not evident in most studies. However studies in Colombia [19], and Kazakhstan [28] show a higher prevalence among younger patients.

EBV has two major types, type 1 and type 2 that differ in their capacity to transform B-lymphocytes into a state of continuous proliferation [39]. Type 1 is predominant in Western and Asian countries while B type is predominant in Africa and is seen more commonly in immunosuppressed patients [40,41]. Besides, there are three other variants, which are distinguished by restriction fragment length polymorphism (RFLP) using BamHI and XhoI restriction endonuclease enzymes. At the BamHI-F region Prototype F has the worldwide distribution and 'f' variant which has an extra site for enzyme is predominant in southern China and is associated with nasopharyngeal carcinoma [42,43]. Polymorphism in BamHI-W1/I1 boundary region leads to two variants; type I and 'i'. Type I which is predominant in Japan and China has no site for BamHI enzyme [44,45] and type 'i' with an extra BamHI site is seen more commonly in Western countries [46]. Finally, polymorphism in XhoI restriction site in axon 1 of the LMP1 gene defines XhoI^+ ^which is more prevalent in Western countries [47] and XhoI^-^, which is frequently seen in Asia [44].

Iran is one of the countries which have a high incidence of gastric carcinoma with an annual incidence of 26.1 per 100,000 for males and 11.1 for females. It is the second most prevalent cancer and the first leading cause of cancer-related deaths in men in Iran [48]. However, there is no study about the prevalence or genotype of EBV in gastric cancer in this area. Therefore, we decided to evaluate it in the oldest referral center for gastric cancer in Iran.

## Methods

### Specimens

We examined 273 surgically resected gastric cancer cases in this study. The cases were randomly selected from 1325 gastric cancer cases registered in cancer institutes of Tehran University between 1969 – 2004 which have intact paraffin blocks and pathologic reports. Formalin fixed paraffin-embedded tissue from these cases were selected and sent to Kagoshima University Graduate School of Medical and Dental Sciences, Japan, for further molecular evaluations. Pathologic data were obtained with re-evaluation of H&E stained slides by two pathologists (AA & SGS). All available demographic data were obtained from the available pathologic and medical reports of the patients.

### Pathology

All the specimens were re-classified in regard of predominant histological pattern as intestinal and diffuse type according to Lauren classification [49] and sub-classified according to the guidelines of the Japanese research society for gastric cancer [50] to well differentiated tubular adenocarcinoma (tub1), moderately differentiated adenocarcinoma (tub2), solid poorly differentiated adenocarcinoma (por1), non-solid poorly differentiated adenocarcinoma (por2), signet ring cell carcinoma (sig), mucinous carcinoma (muc) and poorly differentiated lymphoepithelial-like carcinoma (LE).

The tumors location according to predominant location of the lesions were classified as upper third or cardia, middle third or body and lower third or antrum according to the guidelines of the Japanese research society for gastric cancer [50]. There was no multifocal case in our study.

The depth of invasion was classified as mucosal, sub-mucosal, muscularis propria and serosal involvement.

### In situ hybridization

EBV was identified by the expression of EBV-encoded small RNA-1 (EBER-1). In situ hybridization with a complementary digoxigenin-labeled 30-base oligomer was used to detect the EBER-1 according to the procedure previously described by Chang et al [51]. 4–5-μm sections mounted on silane-coated glass slides were prepared for each case. The tissue on the slides was deparaffinized, rehydrated, predigested with pronase, prehybridized, and then hybridized overnight at 37°C with 0.5 ng digoxigenin-labeled probe. The hybridization signal was detected by an antidigoxigenin antibody-alkaline phosphatase conjugate. Sections from a patient with known EBER-positive gastric carcinoma were used for a positive control, and sense probe to EBER-1 was used for a negative control for each procedure.

### Preparation of DNA

The formalin-fixed and paraffin-embedded specimen was cut into 10 μm thick slices, and DNA sample was prepared following the method described by Greer et al [52]. Briefly, the slices were treated with xylene and ethanol and centrifuged at 22,000 g for 20 min, and the resulting pellet was resuspended in 100 μl of digestion buffer (1 M Tris, pH 8.0, 50 mM EDTA, 0.5% Tween 20) with 200 μg/ml Proteinase K and incubated at 55°C overnight. After 10 min boiling at 100°C, extraction and precipitation of DNA was carried out with phenol-chloroform and ethanol, respectively.

### Primers and probes

Four different regions, EBNA-3C, BamHI-F, BamHI-W1/I1, and XhoI site in LMP1, were used to determine viral genotypes. Table 1 shows the list of primer sets and probes used in the present study.

**Table 1:** List of primers and probes used in this study

Genotype	Sequence	Type by probe or size after RE^1 ^digestion	Reference
EBNA-3C			
Primers			
Sense	5'-AGAAGGGGAGCGTGTGTTGT-3'		
Antisense	5'-GGCTCGTTTTTGACGTCGGC-3		Sample et al. (53)
Probes			
Type 1	5'-GAAGATTCATCGTCAGTGTC-3'	153 bp^2^	
Type 2	5'-CCGTGATTTCTACCGGGAGT-3'	246 bp	
BamHI-F			
Primers			
Sense	5'-TCCCACCTGTTACCACATTC-3'	Prototype F: 198 bp	Lung et al. (54)
Antisense	5'-GGCAATGGGACGTCTTGTAA-3'	Variant "f": 127 + 71 bp	
Probe	5'-AAGGCTACCGTGCTAATTACCTCC-3'		Hudson et al. (55)
BamHI-W1/I1			
Primers			
Sense	5'-ACCTGCTACTCTTCGGAAAC-3'	Type I: 205 bp	Lung et al. (54)
Antisense	5'-TCTGTCACAACCTCACTGTC-3'	Type "i": 130+75 bp	
XhoI site in LMP1			
Primers			
Sense	5'-AACAGTAGCGCCAAGAGGAG-3'	XhoI+:113 bp	Sandvej et al. (56)
Antisense	5'-ATGGAACACGACCTTGAGAGG-3'	XhoI-: 67+46 bp	

^1^Restriction enzyme; ^2^Base pairs

Types 1 and 2 were recognized by amplification of U2 region of EBNA-3C gene by primers described by Sample et al [53] which yielded 153 and 246-bp fragments, respectively. They are recognized by Southern blot hybridization (SBH) with type-specific internal probes described by Sample et al [53]. The BamHI-F region was amplified by the primers set described by Lung et al [54]. The consequent 198-bp fragment was digested by BamHI restriction enzyme which yelled a 198-bp fragment in the case of wild type F and 127-bp and 71-bp fragments in the case of 'f' variant. The wild-type F and the f variants were confirmed by SBH with the internal probe as described before [55]. To distinguish I/i variants, the BamHI-W1/I1 region was amplified using the primer set described by Lung et al [54]. Digestion with BamHI restriction enzyme yelled 205-bp fragment in type I and 130-bp and 75-bp fragments in the case of 'i'. They were also confirmed by SBH with a cloned BamHI-I DNA fragment probe. Analysis of polymorphism in exon 1 of LMP1 gene was performed using a primer set described by Sandvej et al [56] which resulted 113-bp fragments. After digestion with XhoI restriction enzyme, XhoI^+ ^cases which contain XhoI cleavage site (XhoI kept) show 67-bp and 46-bp fragments but XhoI^- ^cases show the original undigested 113-bp PCR product. The 113 bp fragment of PCR product of B95-8 cell line was used as the probe to confirm XhoI cleavage site of LMP1 by SBH [57].

For type 1, wild type F, type I, and XhoI^- ^viruses the B95-8 cell line was used as positive control. The cell lines AG786 and Akata and the cloned BamHI-'f' and BamHI-"i" DNA fragments served as positive controls for type 2, XhoI^-^, "f" variant, and type "i" viruses, respectively. Human herpes virus 6 infected cell line MOLT-4 used as negative control [58].

### Polymerase chain reaction

Amplification of target DNAs was carried out using 2 μl of DNA and mixture of 10 mM Tris-HCl, pH 8.0, 50 mm KCl, 1.5 mM MgCl_2_, 200 μM dNTPs, 1 μM of each primer and 1.0 U Taq Polymerase (Hot Star, Qiagen, Germany) in a final volume of 25 μl mixture. The protocol used for amplification of EBNA-3C gene was one cycle at 95°C for 15 min, followed by 40 cycles of denaturation at 95°C for 1 min, annealing at 46°C for 1 min and elongation at 72°C for 1 min and finally ended with 5 min at 72°C. The protocol used for BamHI-F, BamHI-W1/I1 and XhoI site amplification was one cycle at 95°C for 5 min followed by 40 cycles of denaturation at 95°C for 1 min, annealing at 43°C for 1 min and elongation at 72°C for 1 min and finally ended with 5 min at 72°C. Electrophoresis in a 2% agarose gel and staining with 0.5 μg/ml of ethidium bromide was carried out to identifying the PCR products.

### Restriction fragment length polymorphism analysis

Amplified DNA products (15 μl) were digested with 10 U of BamHI or XhoI restriction enzymes, according to manufacturer's instruction and visualized by electrophoresis in a 2% agarose gel stained with 0.5 μg/ml of ethidium bromide and transferred onto a nylon membrane for SBH.

### Southern blot hybridization

Specificity of PCR reaction was confirmed by SBH. The electrophoretic DNA was transferred onto a Hybond N+ nylon membrane (Amersham, Pharmacia Biotech, UK) by capillary blotting using 0.4 N NaOH solution. Membranes were prehybridized with hybridization buffer for 1 h at 42°C. After adding the probe, hybridization was carried out overnight at 42°C. Probes of types A and B, and BamH-I F were labeled with Dig oligonucleotide 3'-end labeling kit and detected by Dig luminescent detection kit (Boehringer Mannheim, Germany). For detecting the BamHI-I fragment and XhoI polymorphism in LMP1, hybridization was carried out using the ECL direct labeling and detection kit (Amersham, Pharmacia Biotech, UK) according to the manufacturer's instructions.

### Statistical analysis

Statistical analysis was done using LogExact 7.0 software (a statistical package for regression procedure featuring exact method); Cytel Studio 7.0.0 package (Cytel Software Corporation, 2005). Exact test of binary logistic analysis was conducted to compare the proportion of EBV positive cases among gastric adenocarcinoma. Sex, age groups (<50, 50–69 and ≥ 70 years), anatomical location of tumor (antrum, body and cardia), invasion of tumor (serosa, others) and Lauren's histological classification were included as independent variables. Multinominal variables were divided to *n-1 *dummy binominal variables (*n *was the number of categories). Odds ratio with 95% confidence interval was calculated for each category. Type 1 error (α) was set at 0.05. All p values were two-sided.

## Results

We examined 273 gastric cancers including 196 (72%) males and 77 (28%) females. The mean age was 57.3 ± 11.3 (SD) years (range: 24–90; median: 58). Most of them (108 cases; 66%) were between 50 to 69 years, 54 (20%) cases were under 50 and the remaining were over 70 years. One hundred thirty cases (48%) were located in antrum, and 64 (23%) and 71 (26%) cases were located in body and cardia, respectively. In 5 cases the location was not defined and in 3 cases all the gastric wall were involved (linitis plastica) which were not included in statistical analysis. As shown in table 2, most of the cases were intestinal type (56%).

**Table 2:** Distribution of EBER-1 positive gastric carcinomas by histological type and gender

Histology^1^	Male	Female	Total
			
	EBER+/N^2 ^(%)	EBER+/N (%)	EBER+/N (%)
**Intestinal**	**1/113 (1)**	**0/39 (0)**	**1/152 (1)**
pap	0/11 (0)	0/3 (0)	0/14 (0)
tub1	0/36 (0)	0/11 (0)	0/47 (0)
tub2	1/50 (2)	0/16 (0)	1/66 (1)
muc	0/16 (0)	0/9 (0)	0/25 (0)
**Diffuse**	**7/83 (8)**	**1/38 (3)**	**8/121 (7)**
por1	3/34 (8)	1/16 (6)	4/50 (8)
por2	4/40 (10)	0/18 (0)	4/58 (8)
sig	0/9 (0)	0/4 (0)	0/13 (0)

^1 ^pap:papillary adenocarcinoma; tub1:well differentiated tubular adenocarcinoma; tub2:moderately differentiated adenocarcinoma; por1:solid poorly differentiated adenocarcinoma; por2:non-solid poorly differentiated adenocarcinoma; sig: signet ring cell carcinoma; muc:mucinous carcinoma; ^2 ^total number of cases in each group (Based on Japanese research society for gastric cancer)

### Prevalence of EBV positive cases

**Figure 1:**
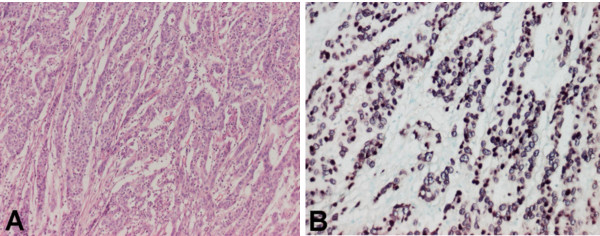
**A case of EBV positive gasrtric carcinoma**. Moderately differentiated tubular adenocarcinoma with occasional tubul formation (A); nuclear EBER positive signal in all tumoral cells detected with in situ hybridization (B).

EBER-1 was detected in nine out of 273 cases (3%) which were considered as EBV-GC. The signal was restricted only to the nuclei of tumoral cells but not found in non-tumoral cells (Fig. 1).

### Factors associated with EBV positive cases

Table 3 shows the frequencies of EBV-GC by age, gender, location, histology and invasion as well as the result of logistic analysis.

**Table 3:** Result of logistic analysis

Variables	EBER+/N^1^	(%)	OR^2^	95% CI^2^
Gender				
Female	1/76	(1)	1	reference
Male	8/188	(4)	0.3	0.01–2.96
		*P *= 0.595		
Age				
≥ 70	1/36	(3)	1	reference
50–69	5/180	(3)	0.8	0.08–44.62
<50	3/54	(6)	1.3	0.08–81.25
		*P *for trend = 1.0		
Location				
Upper	1/71	(1)	1	reference
Middle	5/64	(8)	6.0	0.1–77.86
Lower	3/130	(2)	1.4	0.63–296.7
		*P *for heterogeneity = 1.64		
Histology				
Diffuse	8/121	(7)	1	reference
Intestinal	1/152	(1)	0.08	0.002–0.64
		*P *= 0.009		
Invasion				
Muscle	3/33	(9)	1	reference
serose	6/222	(3)	3.6	0.47–24.03
		*P *= 0.243		

^1^Total number in each group; ^2^OR and 95% confidence interval were obtained from exact test of binary logistic analysis.

#### Gender and age

Eight out of 188 (4%) male cases and 1 out of 76 (1%) female cases were EBV positive. Although there was a male predominance in this study, it was not statistically significant (*p *= 0.5). There was also no relationship between age and EBV-GC (*p *= 1).

#### Tumor location

According to anatomic location, 5 out of 64 (8%) body tumors, 1 out of 71 (1%) cardiac tumors and 3 out of 130 (2%) antral tumors were EBV associated. Although most of EBV-GC were located in upper two-thirds, it was not significant statistically (*p *= 0.1).

#### Histological type

Based on Lauren's classification, 8 out of 121 (7%) diffuse type cases were EBV associated but only 1 out of 152 intestinal type was EBV positive, which was statistically significant (*p *= 0.009). According to Japanese classification, por2 and por1 were the predominant histologic types in EBV-GC (Table 2), but regarding the low number of cases in each group statistical analysis could not be performed.

#### Invasion

In this study there were only 2 cases which were limited to submucosa and none of them shows EBER-1 signal in ISH. Thus, considering of the low number, these cases were not considered in statistical analysis. There was no difference between EBV positive and negative cases regarding invasion to serosa and muscularis propria (*p *= 0.24).

#### Other findings

The noteworthy point in our study was the fact that 6 out of 9 EBV-GCs patients were born between "1928–1930". However, the statistical analysis was not made because of the low number.

### Genotyping

**Figure 2:**
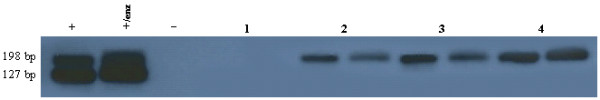
**Result of southern blot hybridization for BamHI-F region**. Four specimens are seen in this figure, each sample was loaded in 2 lanes with and without enzyme, respectively from left. The first specimen was not amplified in this experiment. The other three specimens were not cleaved by Bam-HI enzyme which means they are wild-type F. (+: positive control; +/enz: positive control with enzyme; -: negative control).

**Figure 3:**
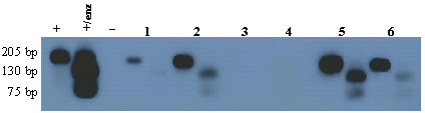
**Result of southern blot hybridization for BamHI-W1/I1 region**. Each sample was loaded in 2 lanes with and without enzyme, respectively from left. In sample one there is no band in the second lane which suggests that the PCR product was digested but we could not detect the fragmented bands since the original band contained small number of DNA copies, so we regarded it as type "i". Samples 2, 5, and 6 are cleaved which means they are type "i". Polymorphism of samples 3 and 4 were not determined in this experiment. (+: positive control; +/enz: positive control with enzyme; -: negative control).

**Figure 4:**
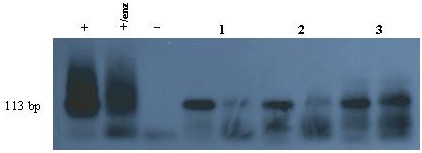
**Result of XhoI polymorphism in southern blot hybridization**. Each sample was loaded in 2 lanes with and without enzyme respectively from left. The first and the second specimens are cleaved with enzyme which means they are XhoI+. The third one is XhoI-. (+: positive control; +/enz: positive control with enzyme; -: negative control).

The subtype of EBV genome was determined in all 9 cases. All of our cases were type 1. The BamHI-F region was amplified successfully in 8 out of 9 of EBV-GCs, which six of them showed prototype F (Fig. 2). Amplification of BamHI-I/WI1 was successful in all of the cases and, except one; all the cases were "i" type (Fig. 3). Analysis of polymorphism in exon 1 of LMP1 gene revealed that 3 cases were XhoI^- ^and 6 cases were XhoI^+ ^(Fig 4). Table 4 summarized all the demographic, pathologic and genotypic data of the EBV-GC in this study.

**Table 4:** Clinico-pathologic and genetic characteristics of EBV-GC

	Birth year	Diagnostic year	Age	Gender	Lacation	Invasion	Histology Lauren/WHO	EBNA-3 type A/B	BamHI-F type F/"f"	BamHI-I type I/"i"	XhoI +/-
1	1949	1981	32	Male	Lower	Muscle	Diffuse/por1	A	F	"i"	+
2	1929	1982	53	Male	Middle	Muscle	Diffuse/por1	A	F	"i"	-
3	1928	1983	55	Male	Middle	Serosa	Intestinal/tub2	A	F	"i"	+
4	1928	1983	55	Male	Middle	Serosa	Diffuse/por2	A	F	I	+
5	1940	1983	43	Female	Lower	Muscle	Diffuse/por1	A	F	"i"	-
6	1930	1986	56	Male	Middle	Serosa	Diffuse/por2	A	ND^1^	"i"	+
7	1928	1988	60	Male	Upper	Serosa	Diffuse/por1	A	F	"i"	+
8	1928	2001	73	Male	Middle	Serosa	Difuse/por2	A	"f'	"i"	-
9	1953	2002	49	Male	Lower	Seroasa	Diffuse/por2	A	"f"	"i"	+

^1^Not determined; tub2: moderately differentiated adenocarcinoma por1: solid poorly differentiated adenocarcinoma; por2: non-solid poorly differentiated adenocarcinoma

## Discussion

The present study detected EBV-GCs in 3% of gastric carcinomas and suggested that the majority of gastric carcinomas in Iran are EBV-negative. There were no LELCs, which are well known to be strongly associated with EBV [59]. Lace pattern, which is a unique morphology in EBV-positive early gastric carcinoma [60], was not seen in our study. This frequency is among the lowest frequencies in the world. As mentioned above, according to WHO classification, the north of Iran is considered as one the regions with high gastric carcinoma frequency in the world [61]. The sparse studies in Iran also show this fact except that they show more or less homogeneity throughout the country [48,62,63]. Thus, the low EBV-GC frequency in Iran supports the previous hypothesis that high risk countries for GC have low rates of EBV-GC [64]. This also confirms the belief that it is low in Asia as the prevalence of 3% in Korea [25], 6% in Japan [24], 6% in china [26], 5% in India [18], 2% in Pakistan [29] and maximal 10% in Malaysia [27], Taiwan [21] and Kazakhstan [28]. However, this is much lower than Kazakhstan frequency, (our nearby country) with many similarities in the custom and socioeconomic conditions.

The prevalence of EBV-GC in diffuse type gastric carcinoma was more in our study, a fact which was seen in most of studies of Latin America [16,32] and some countries in Asia such as Korea [25], China [26] and India [18]. However, there are many contradictory studies which did not show any relationship in this concept [15,17,19,23,33]. Koriyama et al [23] in their classic study proposed that there are many other factors which have effect on the incidence of diffuse type gastric cancer such as age, gender and location and the conflicting results in different studies could be explained by the difference in distribution of these factors.

Although the ratio of male/female was approximately 4 in our study, it was not significant in statistical analysis. The absence of gender preference was seen in other studies in Mexico [32,37] Chile [16] and one area in China [38]. However, we should consider that the low number of positive cases in our study might be the reason of insignificant result in the analysis. Meanwhile, Koriyama et al [23] show that the gender preference for EBV is restricted to diffuse type GC and is not seen in intestinal type. Nevertheless, regarding the low number of our cases, we could not perform the analysis separately for each type.

Increase in cancer of upper portion of the stomach despite decrease in total incidence of gastric cancers is seen in many studies around the world [65,66]. There are also many evidences that this type of gastric cancer has different risk factors [67-69]. EBV is one of them [15-31]. Abdirad et al [70] also show that there is such increase in cancer of upper third of stomach in Iran. Yet, despite our expectation, this study did not show any preference of EBV for upper or middle third of stomach. It is important to notice that in spite of many confirmatory studies in this context, there is also no such relation in our nearby countries, i.e. Kazakhstan [28] and India [18].

EBV in our study did not show any relation with age and depth of invasion. Some studies indicate that there is tendency of EBV-GC to occur in lower age [19,26,33,38]. However, Koriyama et al [23] revealed that only intestinal type of EBV-GC shows this age preference, and proposed that difference in age of exposure to specific cofactors for each of these types is the probable reason.

One of the interesting findings of the present study is the fact that 6 out of our 9 EBV-GC cases which were selected randomly, were born during the period between 1928 and 1930. Although we could not perform any analysis in regard of the low number of positive cases, this finding seems important since an epidemiological study suggested that EBV infection at early childhood may be related to the development of EBV-GC in adulthood [71]. Although we have no evidence, some hypothesis could be considered. For example, specific conditions or events, leading to EBV infection in early childhood or resulting to exposure to some specific co-risk factors which might have existed around the late 1920s. Therefore, we propose a case control study focusing on gastric carcinoma in patients born around the late 1920s to compare the prevalence of EBV in these patients with the others.

Genotyping of the virus revealed that type 1 is the exclusive type in all of our EBV-GC. The predominance of type 1 is in agreement with other studies [18,45,58,40]. Type 2 is mostly seen in Africa [40] and there is a belief that it is weaker than type 2 and is mostly seen in immunodeficient patients [40,72]. The prototype F at BamHI-F was the most common type in this study. This type has a worldwide distribution except in southern China which "f" variant is prevalent and causes nasopharyngeal carcinoma [43,45]. Our finding is the same as similar studies in gastric cancers in India [18], Japan [73], and Chile [58]. BamHI-W1/I1 region polymorphism analysis shows that 90% of our cases were type "i". Previous studies revealed that type I is the most prevalent in Asia [45,54] and type "i" is the most in western countries [46]. This pattern of distribution had been also seen in similar studies on EBV-GC [18,45,73]. However, our finding seems to be similar to western countries like Corvalan findings in Colombia and Chile [58]. XhoI^+ ^was the most prevalent type at XhoI restriction site in exon one of LMP1 gene in the present study. This finding is also similar to Corvalan finding in EBV-GC in Colombia and Chil [58] and in contrast to findings in eastern Asia [44].

The most important finding in this study was the combination of "i"/XhoI^+ ^in 6 of our cases. Corvalan et al had shown that this combination is significantly more in EBV-GC than healthy people [58]. They propose that the existence of certain gene with transforming capacity in the vicinity of these sites is the probable explanation of more tumorigenecity of this variant. This finding is against the belief that EBV strains are geographically distributed but not disease restricted [73]. However, we need first to evaluate the genotype of EBV in healthy people in Iran before any conclusion. To our knowledge, the genotype distribution among healthy population in Iran is not reported in the literature.

## Conclusion

In summary, we could say that our study is the first to describe the frequency of EBV-GC in Iran and in the Middle East, highlighting a very low prevalence. The most important feature of the present study is the fact that we could use the cases diagnosed as early as 1960. Such a feature made it possible to examine the effect of birth year. Interestingly, EBV-GC in the present study was dominated by the patients born during the period between 1928 and 1930. Most of other findings such as diffuse histology, weak male predominance and genotype of the cases were similar to studies performed in Latin America.
